# The diagnosis and management of patients with idiopathic osteolysis

**DOI:** 10.1186/1546-0096-9-31

**Published:** 2011-10-13

**Authors:** Ali Al Kaissi, Sabine Scholl-Buergi, Rainer Biedermann, Kathrin Maurer, Jochen G Hofstaetter, Klaus Klaushofer, Franz Grill

**Affiliations:** 1 Ludwig-Boltzmann Institute of Osteology at the Hanusch Hospital of WGKK and AUVA Trauma Centre Meidling, First Medical Department, Hanusch Hospital, Vienna, Austria; 2 Innsbruck Medical University, Department of Paediatrics IV, Neonatology, Neuropaediatrics and Inherited Metabolic disorders, Innsbruck, Austria; 3 University Clinic for Orthopaedic Surgery, Innsbruck, Austria; 4 Innsbruck Medical University, Department of Radiology, Innsbruck, Austria; 5 Department of Orthopaedic Surgery, Vienna General Hospital, Medical University of Vienna, Vienna, Austria; 6 Orthopaedic Hospital of Speising, Paediatric Department, Vienna, Austria

**Keywords:** Gorham-Stout disease, Angiomatosis, Nephropathy, Winchester syndrome, Histology, Genetics, 3-Methylcrontonyl CoA Carboxylase deficiency

## Abstract

Idiopathic osteolysis or disappearing bone disease is a condition characterized by the spontaneous onset of rapid destruction and resorption of a single bone or multiple bones. Disappearing bone disorder is a disease of several diagnostic types. We are presenting three patients with osteolysis who have different underlying pathological features. Detailed phenotypic assessment, radiologic and CT scanning, and histological and genetic testing were the baseline diagnostic tools utilized for diagnosis of each osteolysis syndrome. The first patient was found to have Gorham-Stout syndrome (non-heritable). The complete destruction of pelvic bones associated with aggressive upward extension to adjacent bones (vertebral column and skull base) was notable and skeletal angiomatosis was detected. The second patient showed severe and aggressive non-hereditary multicentric osteolysis with bilateral destruction of the hip bones and the tarsal bones as well as a congenital unilateral solitary kidney and nephropathy. The third patient was phenotypically and genotypically compatible with Winchester syndrome resulting in multicentric osteolysis (autosomal recessive). Proven mutation of the (MMP2-Gen) was detected in this third patient that was associated with 3MCC deficiency (3-Methylcrontonyl CoA Carboxylase deficiency). The correct diagnoses in our 3 patients required the exclusion of malignant osteoclastic tumours, inflammatory disorders of bone, vascular disease, and neurogenic arthropathies using history, physical exam, and appropriate testing and imaging. This review demonstrates how to evaluate and treat these complex and difficult patients. Lastly, we described the various management procedures and treatments utilized for these patients.

## Introduction

The inherited osteolysis disorders represent a group of rare diseases characterized by destruction and resorption of affected bones with subsequent skeletal deformities and functional impairment. Previous studies showed unifocal and multicentric osteolyses, autosomal dominant and recessive inheritance, associated with nephropathy, and mental retardation[1]. Hardegger et al [1] described the most commonly accepted classification:

1) Type 1, hereditary multicentric osteolysis with dominant transmission;

2) Type 2, hereditary multicentric osteolysis with recessive transmission:

3) Type 3, nonhereditary multicentric osteolysis with nephropathy;

4) Type 4, Gorham-Stout syndrome;

5) Type 5, Winchester syndrome defined as a monocentric disease of autosomal recessive inheritance.

Another approach is the international Skeletal Dysplasia Registry which classified these disorders into four groups according to their clinical and radiographic criteria and mode of inheritance [2].

Gorham and Stout [3] emphasized the following clinical features of osteolysis syndromes: Progressive osteolysis of one or more bones in children and young adults, history of minor trauma, often associated with a pathological fracture, and vascular malformations in the affected bones or surrounding soft tissues. Patients generally present with bony deformity, with corresponding muscular weakness and localized pain. They suggested that the massive osteolysis results from angiomatosis within the involved bones and the surrounding soft tissue [4-6]. Renal involvement is more severe and occurs more frequently in the type 3 of Hardegger classification [1]. A congenital solitary functioning kidney is part of the spectrum of congenital anomalies of the urinary tract, which is the major cause of end-stage renal failure in children [7,8]. Among the autosomal recessive disorders with predominant multicentric carpal, tarsal, and interphalangeal involvement is Winchester syndrome [9]. The aim of this article is to compare the clinical history, phenotypic, and radiographic changes of idiopathic osteolysis syndromes in three unrelated children.

## Methods

The study protocol was approved by the Medical University of Vienna (Ethics Committee, EK Nr. 921/2009), and informed consent was obtained from the patient's guardians. Two patients were of Austrian origin and one patient was from North Africa. These patients' records were reviewed in the Osteogenetic Department of the Orthopaedic Hospital of Speising, Vienna. Extensive chart and imaging review was performed to prepare this case series illustrative of the spectrum of osteolysis syndromes.

### Patient I

**Figure 1:**
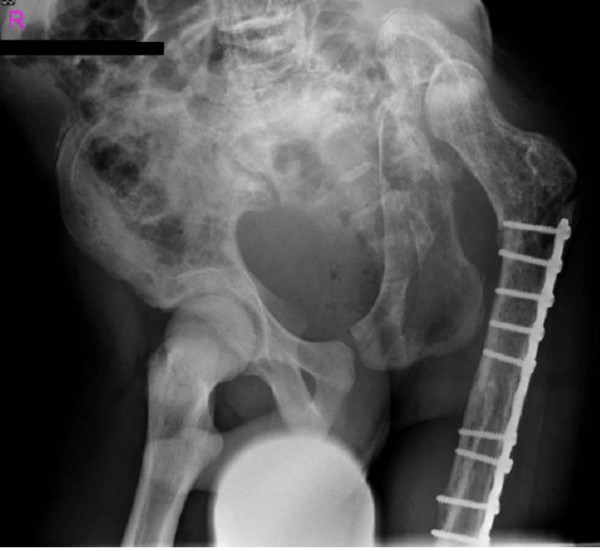
**(Patient I)**. Anteroposterior pelvis radiograph showed complete dissolution of the left hemipelvis, with superior migration of the femoral head. At this stage we fixed the fracture with a long Gamma nail.

**Figure 2:**
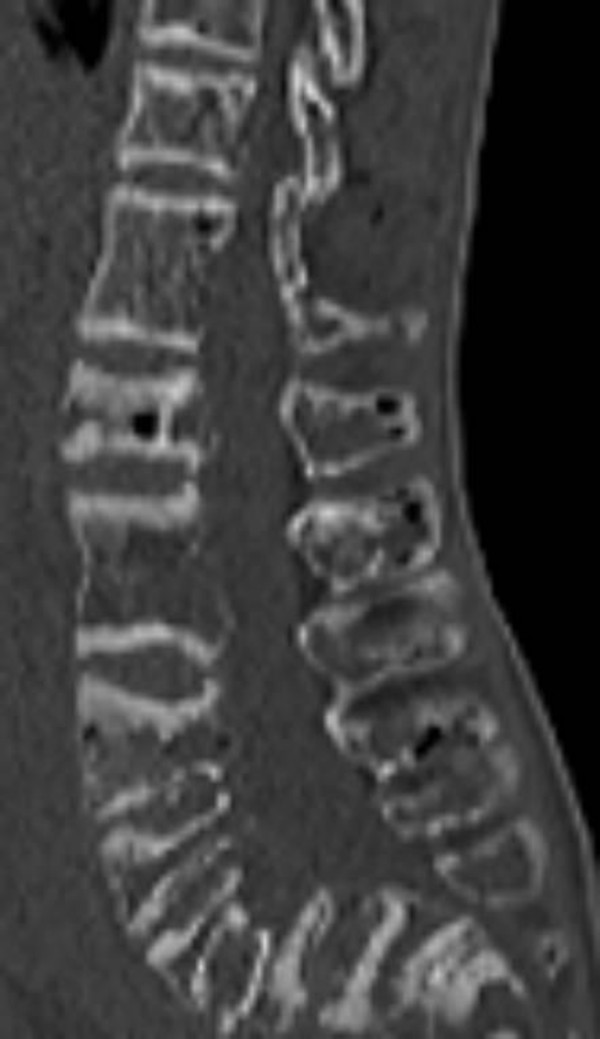
**Sagittal 3DCT scan of the thoracolumbar radiograph showed a combination of deformities ranged from severe flattening, fusion, shrinkage and compression fractures**. A feature mimicking anisospondyly in skeletally dysplastic patients (Different abnormal shapes of the vertebral bodies) was evident.

**Figure 3:**
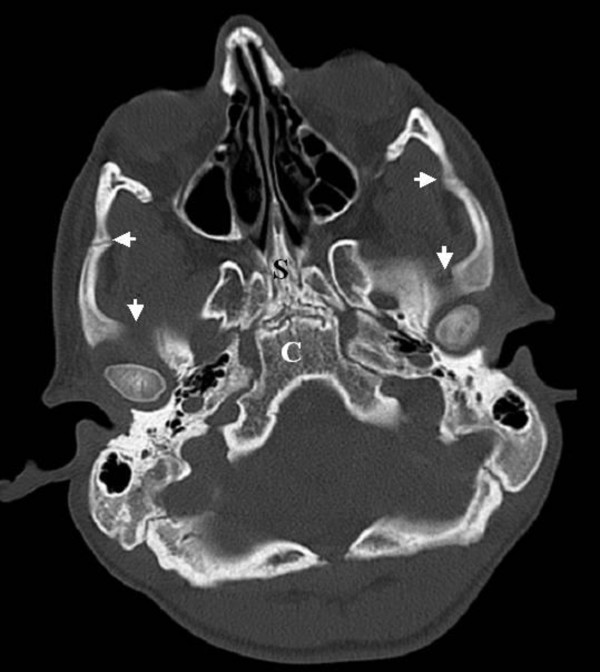
**Axial 3DCT scan of the skull base showed osteolytic destruction of the most skull base bony elements, namely, the squamous part of the temporal bones associated with lysis of the zygomatic arch bilaterally (small arrows)**. S is the sphenoid bone and C is the occipital part of the clivus (both manifested osteolytic destruction associated with significant irregularities). In addition the destruction involved the greater wings of the sphenoid bone (fragmentations and massive irregularities were present).

**Figure 4:**
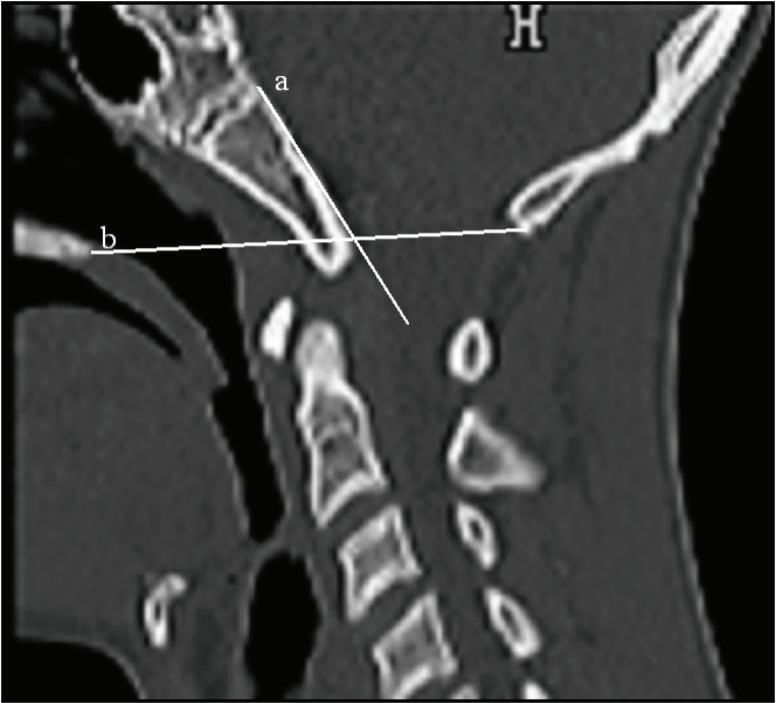
**Sagittal skull base-C1/2 3DCT scan showed significant downward displacement of the clivus onto the foramen magnum**. Wachenheim- Clivus line* was extremely deviated from its norms (line-a). McGregor line** was not applicable but it showed a reverse mechanism of eminent brain insult. *This line is drawn down posterior surface of clivus and its inferior extension should barely touch posterior aspect of odontoid tip (this relationship does not change in flexion and extension). If this line runs behind the odontoid, posterior subluxation has occurred.**This line is to assess whether basilar invagination exists. It is usually drawn from posterior hard palate to base of occiput. If the odontoid process is more than 4.5 mm, this reflects basilar invagination. In our patient the line intersects with the tip of clivus (basion) i.e. prolapse of the clivus onto the foramen magnum secondary to osteolysis of the skull base bony components. As our patient presented with a progressive deformity of the craniocervical and the spine, we might refer to a long posterior fusion with rigid instruments, combined with radiation therapy.

The presenting symptoms in the first patient were pain, weakness, and aggressive destruction of the hip joints resulting in severe joint deformation. Later there was also noted extensive lytic changes of the spine and the skull base, utilizing radiographic, CT scan, and histological examinations. It could be demonstrated that this patient manifested massive osteolysis in correlation with vascular proliferation and angiomatosis. Histological study showed ecstatic vessels covered with endothelium resembling hemangioma. He was the product of non-consanguineous couple from Austria. At birth his growth parameters were around the 50th percentile. The family history was non-contributory. Craniofacially, the patient showed a normal phenotype with no associated dysmorphic features. His subsequent course of development has been normal. At the age of 5 years, pain over the pelvis associated with limitations in his daily activities was the predominating clinical feature. At this age, no specific surgical measures have been taken. Analgesics were the only treatment. Later on the osteolysis had extended to involve the left ileum with destruction of the acetabulum associated with further lysis over the shaft of the left femur. As a result of the severe shortening of the left leg, he sustained 3 fractures of his left femur. The first fracture was stabilized by an intramedullary rod; later the rod protecting the femur against fracture was removed. The femur again fractured after a minor trauma and the fracture was stabilized by an AO plate, not a surgery performed at our institution for this indication. Intramedullary fixation, similar to the techniques in patients with osteogenesis imperfecta, may be the preferred approach. Therefore, when this patient was admitted to our institution because of his femoral fracture, complete dissolution of the left hemipelvis was found and the femur on the affected side was higher. The plate was removed- the fracture was exactly localized at the proximal end of the plate - and a long Gamma nail was used to fix the fracture (Figure 1). The patient was able to walk a few days after surgery using crutches with partial weight bearing. Recently, the patient developed neck pain which worsened with movement and radiated to involve the whole back. Conventional radiographs were of limited value. A sagittal 3DCT scan of the thoracolumbar spine showed a combination of deformities ranging from severe flattening, fusion, shrinkage and compression fractures of the vertebrae (Figure 2). A 3DCT scan to assess the craniocervical bony components was done as well. The axial 3DCT scan of the skull base showed osteolytic destruction of the bony elements of most of the skull base, namely, the squamous part of the temporal bones associated with lysis of the zygomatic arch bilaterally (Figure 2-the small arrows-S is the sphenoid bone and C is the occipital part of the clivus). Both areas manifested osteolytic destruction associated with significant irregularities. In addition the destruction involved the greater wings of the sphenoid bone. Fragmentations and massive irregularities were present (Figure 3). Another scan, a sagittal skull base-C1/2 3DCT scan revealed a significant downward displacement of the clivus onto the foramen magnum. The Wachenheim-Clivus line* was extremely deviated from its norms (line-a). The McGregor line** was not applicable but it suggested a reverse mechanism of eminent brain insult (Figure 4).

**Figure 5:**
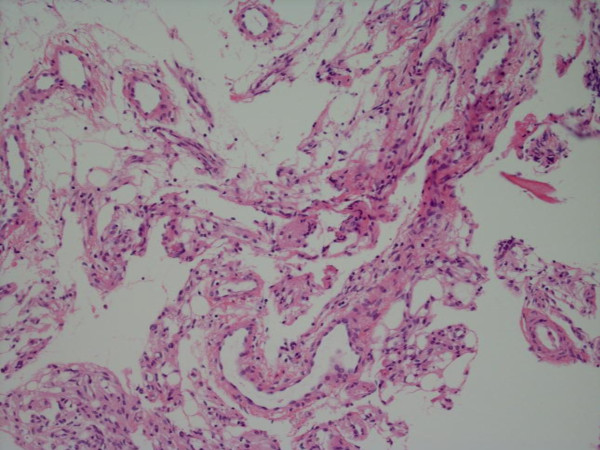
**Specimen obtained at open biopsy of a lesion in the left proximal femur showed multiple, dilated, vascular spaces replacing normal bone marrow elements (H and E, × 100)**.

**Figure 6:**
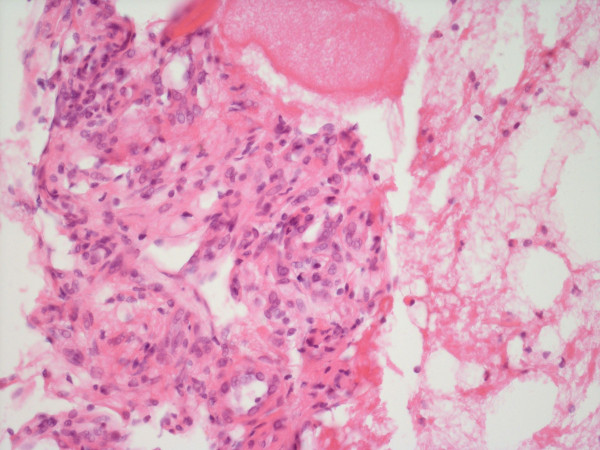
**Note the vascular structure lined by a single layer of endothelial cells at a higher magnification (× 400)**.

The pathological specimen obtained at an open biopsy of a lesion in the left proximal femur demonstrated multiple dilated vascular spaces replacing normal bone marrow elements (H and E, × 100) (Figure 5). Note the vascular structure lined by a single layer of endothelial cells visible at a higher magnification (× 400) (Figure 6). Laboratory investigations showed slight proteinuria and a raised alkaline phosphatase (reflecting active bone turnover). The complete blood count with differential and the blood chemistries were normal. PTH, karyotype, plasma amino acid screening, urine and plasma mucopolysaccharides were all normal. Rheumatologic parameters were normal as well. There are no known definitive treatments for this disorder but various forms of anti-angiogenic, anti-invasive factors and/or drugs are under study. In addition, various forms of chemotherapeutic agents are under study as well. Radiotherapy has been used but often has proven ineffective. This spine disorder is not cured with surgery.

### Patient II

**Figure 7:**
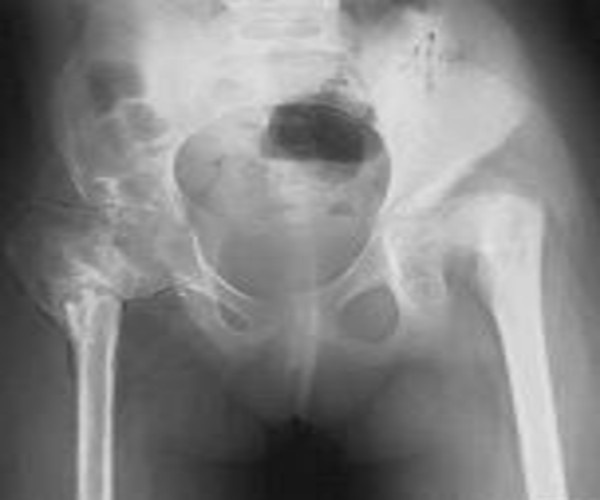
**(Patient II)**. Anteroposterior pelvis radiograph showed massive osteolysis crosses the epiphyseal growth centers of the proximal femora bilaterally, resulting in growth disturbance with radiological appearance resembles the sucked end of a candy-sugar stick, severe osteolysis of the proximal femora were present, the right hip was completely ankylosed with severe generalized osteopenia, progressive osteolysis associated with rudimentary cortices.

**Figure 8:**
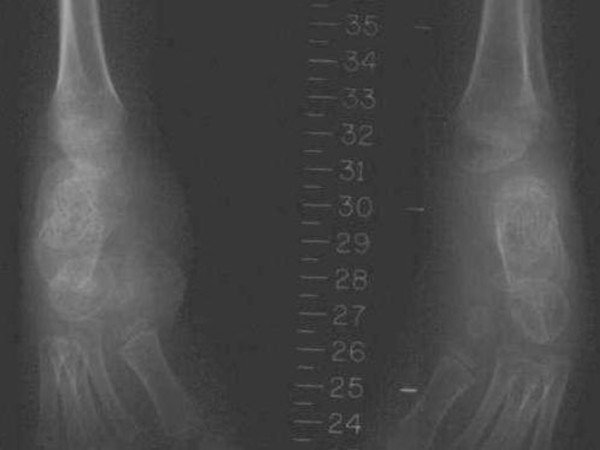
**Anteroposterior radiograph of the feet showed complete resorption of the tarsal bones ended up with subsequent fusion of the melted bones and complete ankylosis with severe osteolysis**.

A 10-year-old girl was referred to our department because of severe pelvic and tarsal pain over the last five years. Our initial evaluation suggested an aggressive multicentric osteolysis involving the hips and tarsal bones that was associated with a nephropathy and a congenital solitary kidney. She was a product of non-consanguineous marriage of North African origin. The family history was non-contributory. Her subsequent course of development has been of moderate motor delay with mild ligamentous hyperlaxity. In infancy a solitary kidney was diagnosed. Clinical examination showed short stature (-3SD). Micrognathia, a short upturned nose and a short philtrum were present. The blood pressure was 190/135 mm/hg. An echocardiogram demonstrated normal heart anatomy. Hearing, vision and intelligence were normal. The hands were small with contractures and the feet were small and deformed. Restriction of movements of the wrists and ankles were evident. Limb length inequality was apparent. Pain over the pelvis associated with limitations in her daily activities was the predominating clinical feature and led to evaluation of the pelvis. The pelvic osteolysis was quite severe, involving the pelvic bones bilaterally. The tarsal bones as well were severely affected. The osteolysis crossed the epiphyseal growth centers of the proximal femurs bilaterally, resulting in growth disturbance. The radiological appearance resembled the sucked end of a candy-sugar stick. Severe osteolysis of the proximal femurs were present. The right hip was completely ankylosed with severe generalized osteopenia and progressive osteolysis was associated with rudimentary cortices (Figure 7). Bilateral tarsal osteolysis has been noted in the right foot; this finding was likely associated with prior episodes of painful swelling of the foot which had been previously diagnosed as due to juvenile rheumatoid arthritis. An anteroposterior (AP) radiograph of the foot showed complete resorption of the tarsal bones. There was subsequent fusion of the melted bones and complete ankylosis due to the severe osteolysis (Figure 8).

At the time as the onset of osteolysis, proteinuria had been detected. Laboratory findings in our evaluation showed a blood urea of 60 mg/dl, a serum creatinine concentration of 1.9 mg/dl, and a creatinine clearance of 57 ml/m/1.73 m2. The serum calcium was 9.7 mg/dl, the phosphorus 5.8 mg/dl, the alkaline phosphatase 218 IU/l, and the PTH (parathyroid hormone) 11.5 pg/ml. A urinalysis was positive for protein and negative for blood, and a 24 hour urine protein excretion was 2.4 g. The parents refused a renal biopsy. The systolic blood pressure was treated with angiotensin-converting enzyme (ACE) inhibitor.

### Patient III

A-5-year-old girl from a consanguineous family (first cousins) presented primarily with progressive contractures of the hands and foot (distal arthropathy). The distal arthropathy was a crippling and painful arthritis with deformity with fusiform swelling of the fingers and a generalized osteopenia. Later she manifested right radial head dislocation and metatarsal fractures. Facial changes were remarkable. Lastly, a mutation of the MMP2 Gene was associated with 3MCC deficiency (3-Methylcrontonyl CoA Carboxylase deficiency was detected.

**Figure 9:**
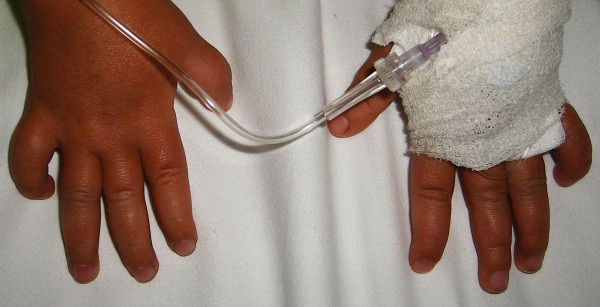
**(patient III)**. Hands photo showed marked decrease range of motion in her wrists and fingers and were mildly puffy. Her fifth finger in particular was noted to be curved like C shape (intermittent polyarthralgia results in progressive joint contractures) associated with swellings in her wrists.

**Figure 10:**
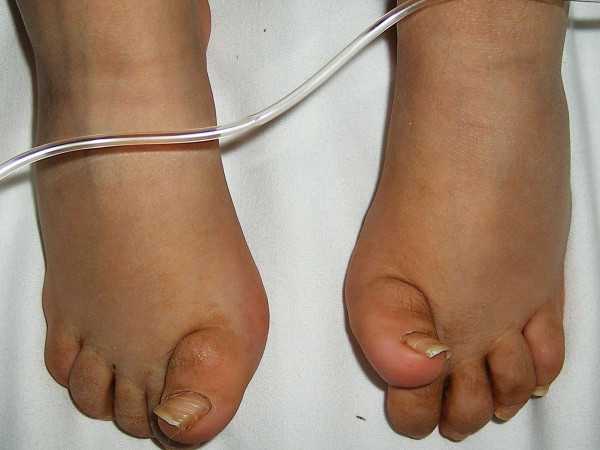
**The extensor tendons in both feet were swollen and her feet showed decreased range of motion in her all toes with eversion of the big toe**.

**Figure 11:**
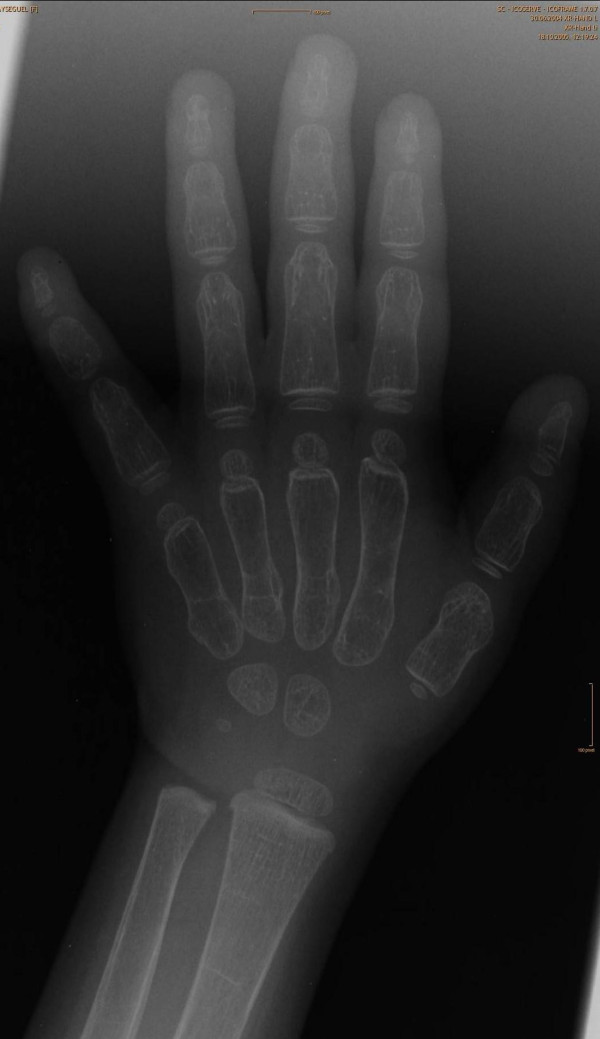
**Anteroposterior hand radiograph at 10 years showed 2.2 years bone age**. Marked widening of the distal portions of the metacarpal metaphyses and diaphyses respectively associated with thinning of the cortices and osteopenia.

**Figure 12:**
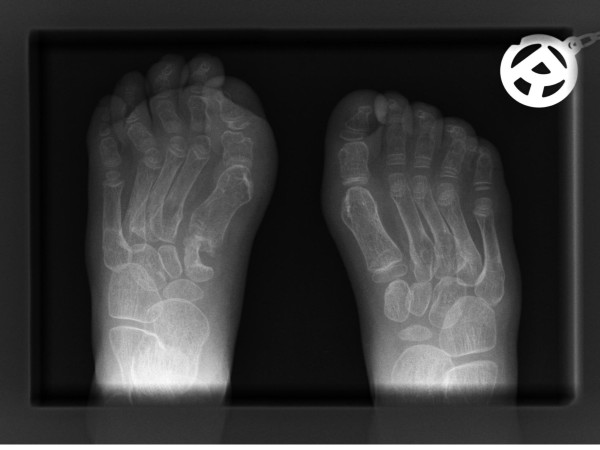
**Anteroposterior radiograph of the foot showed widespread erosions and shortenings of the big toes associated with osteolysis of the tarsals and erosions of the metatarsals and the cortices were thin**.

**Figure 13:**
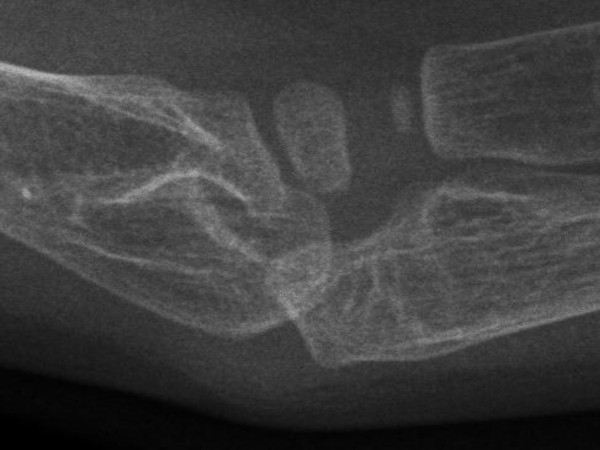
**Osteoporosis of the distal humerus, the radial head, and the olecranon**.

She was a product of uneventful gestation as well as delivery. At the age of 18 months, her parents observed an abnormal gait and an element of contractures appeared. Her contractures were of progressive nature and her gait was characterized by pronation and eversion of her feet associated with edema and limited movements of the interphalangeal joints. Moreover, she was unable to straighten the hands and feet properly. By the age of five years, walking became a burden because of pain in her feet and stiffness. Clinical examination revealed a girl with short stature (-2SD). She had course facies, ptosis, proptosis, a high vaulted palate, micrognathia, large ears and a large, bulbous nose. Skin examination showed no specific stigmata, or any other abnormality. Hearing, vision, and intelligence were normal. Renal ultrasound was normal. Orthopaedic examination showed marked decrease range of motion in her wrists and fingers. Her hands and wrists were mildly puffy. Her fifth finger in particular was noted to have a "C" shape (intermittent polyarthralgia results in progressive joint contractures) associated with mild swelling in her wrists (Figure 9). The extensor tendons in both feet were swollen and her feet showed decreased range of motion in her all toes with eversion of the big toe (Figure 10). Anteroposterior hand radiograph at 10 years showed 2.2 years of bone age. Marked widening of the distal portions of the metacarpal metaphyses and diaphyses was associated with thinning of the cortices and osteopenia (Figure 11). An AP radiograph of the foot showed widespread erosions and shortenings of the big toes as well as osteolysis of the tarsal/metatarsals with thin cortices. Severe osteolysis of the tarsal bones is also seen in (Figure 12). A lateral radiograph of the elbow showed osteoporosis of the distal humerus, the radial head, and the olecranon (Figure 13).

Laboratory evaluation showed an elevated ESR and a negative antinuclear antibody. The complete blood count with differential, calcium, phosphate, alkaline phosphatase, 25-hydroxy vitamin D, PTH, karyotype, plasma amino acid screening, urine and plasma mucopolysaccharides tests were all normal. Renal ultrasound and echocardiogram were normal. The neurologic exam as well as vision, hearing and intelligence testing were normal. The patient suffered from a unilateral dislocation of the radial head with the right elbow held in a 90 degrees flexion. Total range of motion was less than 5 degrees. Treatment consisted of open reduction of the radial head and angulation osteotomy of the ulna when the patient was eight years old. A cast was applied for 6 weeks with subsequent physiotherapy for several months. Two years after the intervention there was no passive movement in the elbow. The diagnosis of "Winchester syndrome" has been confirmed by the demonstration of homozygous mutations in the MMP2 gene. The mutation of the MMP2 gene was associated with 3MCC deficiency (3-Methylcrontonyl CoA Carboxylase deficiency).

## Discussion

Gorham-Stout syndrome [3] refers to a condition mainly of young adults (although onset can be between 18 months and 60 years). The presenting symptom is usually pain in a long bone, the pelvis, thorax or spine. Gorham-Stout disease is an aggressive form of skeletal angiomatosis disease. Radiographs reveal osteolysis of the bone involved. This begins in the subcortical region and may lead to a tapering appearance of the bone and then complete disappearance. Variable absorption begins in one bone and frequently progresses to involve multiple contiguous bones, with joints and intervertebral disks posing no barriers. Its clinical presentation is variable, largely depending upon the site of skeletal involvement. The disease pathophysiology commences with intramedullary and subcortical radiolucent foci resembling patchy osteoporosis. It makes slow, irregular, local progress with a concentric shrinkage of the shafts of the bones. The affected bone disappears more or less completely unless spontaneous remission occurs. The pathological process in Gorham disease may affect the axial skeleton as well.

In the literature, the prognosis is generally considered to be good. However, in spinal or thoracic involvement, life-threatening complications can occur [10]. Management of Gorham-Stout syndrome is also a subject of controversy. Various therapeutic options have been described in the literature and all of them have been disappointing. In the past, different aggressive medical therapies have been attempted to stop the bone resorption. Medications such as androgens, chemotherapy (cisplatin or Actinomycin D), and inhibitors of bone resorption (calcitonin and bisphosphonates) have been tried [11]. In order to classify skeletal angiomatosis into aggressive and non-aggressive types, the bases of their clinical behavior, the natural history of the disease and the pattern of skeletal involvement are to be considered.

Renal agenesis is relatively common malformation, which appears during embryonic development and may be unilateral or bilateral. The latter is incompatible with survival. The etiology of unilateral renal agenesis is heterogenous with environmental and genetic influences. Prenatal factors associated to renal agenesis are diabetes mellitus, alcohol exposure, black race, and young maternal age [12,13]. Szöke et al [14] reported a case of idiopathic osteolysis (ICTO) type III associated with Bartter's syndrome. The pathogenesis of ICTO type III is still unknown. Bennett et al [15] speculated that renal involvement, and possibly osteolysis, results from a primary vascular disease since similar vascular changes have been described in coronary vessels, skin, and the synovial cartilage. Shurtleff et al [16] described hereditary arthritis manifested by clinical symptoms of heat, tenderness, and swelling of the joints in childhood, followed by a period of progressive collapse and osteolysis of the carpal and tarsal bones. Biopsy and other laboratory tests indicate an absence of an inflammatory process. However, arteriolar thickening was found in all tissue biopsied. Hypertension and nephropathy associated with abnormal cellular elements found in a high percentage of the involved patients suggest a systemic disorder manifested primarily by vascular involvement.

Among the autosomal recessive disorders with predominately multicentric carpal, tarsal, and interphalangeal involvement with no other systemic, renal, or neurological abnormalities is Winchester syndrome (WS). Winchester syndrome is a rare autosomal recessive disorder resulting in multicentric osteolysis. Onset of the condition may be towards the end of the first year of life with symmetrical painful swelling of the hands, fingers, wrists and ankles. Intermittent polyarthralgia results in progressive joint contractures. Oval or linear raised areas of thickened skin may appear over the back, flanks and lateral aspects of the arm. These lesions spread to cause leathery, thickened, hypertrichotic, pigmented skin. Other features are corneal opacities appearing in mid-childhood, retarded growth, carpal and tarsal osteolysis and rheumatoid-like destruction of the small joints. It was originally believed that WS was a mucopolysaccharides storage disease [9]. Gingival hypertrophy has been found in 6 patients including the one reported by Sidwell et al [17]. Zankl et al [18] showed that WS is caused by mutation in the gene encoding matrix metalloproteinase-2 (MMP2, collagenase type IV-A), although the precise pathogenesis is unknown. The metalloproteinases are a group of structurally related endopeptidases that require a metal cofactor. They are involved in the breakdown of extracellular matrix and basement membrane components; therefore, they play an important role in connective tissue turnover and bone formation.

3-methylcrotonyl-CoA carboxylase deficiency (3-MCC deficiency) is an inherited disorder in which the body is unable to process certain proteins properly. The enzyme responsible for this condition takes part in the breakdown of leucine and is biotin dependent. It should be noted that some patients with this enzyme deficiency might have a deficiency of all 3 mitochondrial, biotin dependent carboxylase. This includes Propionyl-CoA carboxylase and pyruvate carboxylases as well as the enzyme under consideration. There is a persistent high excretion of 3-hydroxyisovalerate and 3-methylcrotonylglycine, usually combined with a secondary carnitine deficiency. Note that the enzyme is a heterodimer consisting of alpha and beta subunits. Clinically patients with 3-MCC deficiency are presented with hypotonia and episodic metabolic acidosis. Some cases might be thought to have a viral encephalitis [19]. One case reported by Murayama et al. [20] had failure to thrive, had seizures and exhibited chronic progressive rigidity, dystonia and spasticity. She was initially thought to have cerebral palsy. The case reported by Ihara et al [21] was picked up on the neonatal screening programme for maple syrup urine disease. Winchester syndrome has not been reported in any of the above mentioned entities. Both the alpha and the beta subunits of MCC have been mapped: alpha to 3q25-27 and beta to 5q12-13 by Gallardo et al [22]. These authors have found mutations in both subunits. Further mutations in MCCA (3q26-q28) and MCCB (5q13) were reported by Holzinger et al. [23]. No previous reports described the simultaneous mutation of MMP2-Gen and 3-MCC deficiency in patients with Winchester syndrome.

## Conclusions

In all types of idiopathic osteolysis, the exact pathogenetic mechanism remains unknown. The types of osteolysis are heterogeneous and clinically diverse with different genetic and molecular changes. Gorham and Stout [3] suggested that in the presence of a hemangioma, an active hyperemia with proliferation of periosteal capillaries ensues. This distorts the bone turnover balance in favor of osteoclastic resorption. In non-hereditary multicentric osteolysis with nephropathy, it was obvious that at the time as onset of osteolysis, proteinuria has been detected. In our patient with Winchester syndrome, the osteolytic process begun as peripheral arthropathy (carpal and tarsal osteolysis and rheumatoid-like destruction of the small joints) with simultaneous osteolysis of the right elbow causing subluxation and limitation of movement (partial osteolysis of the distal humeral epiphysis, radial head, and the olecranon). The patient's facial appearance was distinctive. The osteolysis was progressive, but neither nodules nor cataracts have been developed in this patient. Finally, the link between WS and 3-MCC deficiency is our patient was difficult to establish and therefore the 3-MCC deficiency may be a separate disorder.

## Consent

Written informed consent was obtained from the patients for publication of this review and accompanying images. A copy of the written consent is available for review by the Editor-in-Chief of this journal

## Competing interests

The authors declare that they have no competing interests.

## Authors' contributions

AAK: drafted the manuscript and analyzed the data. SS-B, RB and KM participated in the design of the third patient. JH and KK participated in coordination of the study. FG conceived of the study, and participated in its design and coordination. All authors read and approved the final manuscript.
